# An ovine model shows that subcutaneous adipose tissue fibrosis occurs early in polycystic ovary syndrome (PCOS)

**DOI:** 10.1530/JME-25-0106

**Published:** 2025-11-19

**Authors:** Yuan Wang, Giovanni Levate, Michael T Rae, W Colin Duncan, Katarzyna J Siemienowicz

**Affiliations:** ^1^ Centre for Reproductive Health, Institute for Regeneration and Repair, The University of Edinburgh, Edinburgh, UK; ^2^ Centre for Biomedicine and Global Health, School of Applied Sciences, Edinburgh Napier University, Edinburgh, UK

**Keywords:** androgen, fat, inflammation, fibrosis, collagen, sheep

## Abstract

Prenatally androgenised (PA) sheep are a clinically realistic model of polycystic ovary syndrome (PCOS). They have dysfunctional subcutaneous adipose tissue (SAT) with reduced adipogenesis in adolescence and enlarged adipocytes with increased inflammation in adulthood. We hypothesised that analysis of SAT in young adults, after adipogenesis is complete but before inflammation is apparent, would give insights into the evolution of adipose tissue dysfunction. Pregnant sheep were treated intramuscularly with 100 mg testosterone propionate or vehicle control (C) twice weekly from day 62–102 of gestation. Weight-matched female offspring (PA = 10; C = 10) were studied up to 22 months of age. Glucose tolerance testing was performed, and at sacrifice SAT was fixed for histological analysis and frozen for RNA sequencing (RNAseq) and gene expression analysis. There was no difference in the average size of SAT adipocytes between PA and C young adults. There were no differences in the expression of the adipogenesis markers *PPARG*, *CEBPA* and *CEBPB*, or the inflammatory markers *TNF* and *IL6*, although PA sheep were already hyperinsulinaemic. RNAseq identified 792 potentially differentially expressed (*P* < 0.05) genes in PA sheep SAT (406 upregulated; 386 downregulated). Ingenuity Pathway Analysis highlighted upregulation of fibrotic pathways in the SAT of PA sheep. *POSTN*, associated with tissue fibrosis, and *COL1A1*, *COL1A2* and *COL3A1* were significantly elevated, and histochemistry showed significantly increased SAT fibrosis in PA sheep. Early fibrotic changes in SAT occur before inflammatory gene expression in PA sheep. A fibrotic barrier to healthy adipocyte expansion may have a mechanistic role in the development of inflammation in PCOS.

## Introduction

Polycystic ovary syndrome (PCOS) is a complex pathological condition associated with reproductive and metabolic dysfunctions, including anovulation, hyperandrogenism, dyslipidaemia, insulin resistance (IR) and obesity (Fauser *et al.* 2012). It occurs globally in 11–13% (Stener-Victorin *et al.* 2024) of women of reproductive age, with some heritability (Balen 2004). Preclinical models and human experience show that gestational exposure to increased androgens results in female offspring with features of PCOS, including increased androgens, IR and obesity (Stener-Victorin *et al.* 2020). As PCOS is a lifelong condition with limited therapeutic options, there is an unmet need for novel interventions. The use of clinically realistic preclinical models is one strategy to increase understanding of PCOS and identify novel therapeutic areas and paradigms (Siemienowicz *et al.* 2020). Although hyperandrogenaemia, menstrual irregularity and anovulatory infertility are characteristic traits of PCOS, it seems that the most concerning long-term key issues are the metabolic problems associated with the syndrome (Fauser *et al.* 2012).

As the metabolic consequences of PCOS are markedly exacerbated by obesity (Hoeger *et al.* 2021), and up to 80% of women with PCOS are overweight or obese (Fauser *et al.* 2012), understanding and targeting obesity in PCOS are critical. Metabolic disturbances of obesity in PCOS are frequently described as relating to visceral fat (Wehr *et al.* 2009), but it is clear that subcutaneous adipose tissue (SAT) dysfunction is also linked to an adverse metabolic environment (de Medeiros *et al.* 2021). Interestingly, the adipose tissue of women with PCOS exhibits enlarged adipocytes, associated with decreased adiponectin production and IR, suggesting that alterations in the function and morphology of adipose tissue contribute to the pathogenesis of PCOS (Echiburú *et al.* 2018). Our group has previously documented that ewes exposed to increased androgens *in utero* manifest ovarian, hormonal and metabolic phenotypes reminiscent of PCOS. Like women with PCOS, the SAT of the PCOS-like sheep is dysfunctional, with larger adipocytes, decreased adiponectin, more inflammatory markers and IR (Ramaswamy *et al.* 2016, Siemienowicz *et al.* 2020, Siemienowicz *et al.* 2021, Levate *et al.* 2024).

In adolescence (11 months of age), female offspring of sheep exposed to increased testosterone during mid-pregnancy have reduced expression of genes and proteins driving adipogenesis in SAT and decreased beneficial adipokines, independent of central adiposity. In the older adult (30 months of age), the structure and function of SAT were altered, with fewer and larger adipocytes, more inflammatory transcripts and less capacity for thermogenesis (Siemienowicz *et al.* 2020, Siemienowicz *et al.* 2021). The evolving dysfunction seen in SAT was not seen in visceral adipose tissue (Levate *et al.* 2024). This suggests SAT-specific dysfunction as the PCOS-like sheep ages, which is likely to play a central role in the metabolic abnormalities observed in PCOS.

We hypothesised that analysis of adipose tissue in young adults, after adipogenesis is completed but before increased inflammatory transcripts occur, would give insights into the evolution of adipose tissue dysfunction in PCOS. Herein, we have investigated SAT structure and function in younger adulthood (22 months of age) in weight-matched female control and PCOS sheep.

## Materials and methods

### Animal treatment and tissue collection

Experiments were conducted under a UK Home Office project licence (PPL60/4401) after ethical committee approval by the Animal Welfare and Ethical Review Body (AWERB) at the University of Edinburgh. Tissue was collected from 22-month-old sheep under natural seasonal breeding conditions, Scottish Greyface ewes were mated with Texel rams. Ewes were managed with progesterone sponges to align oestrous cycles before mating (Siemienowicz *et al.* 2020). Pregnant sheep were treated intramuscularly with 100 mg testosterone propionate (AMS Biotechnology (Europe) Ltd, UK), in vegetable oil and 5% ethanol, twice weekly from 62 to 102 days of gestation (term = 147 days). Controls (C) were treated with the same volume of vehicle without testosterone. The dose and method of testosterone treatment were selected based on published data and our group’s previous research regarding postnatal reproductive disruptions (Birch *et al.* 2003, Hogg *et al.* 2012, Siemienowicz *et al.* 2020). Female lambs were weaned at 3 months of age and fed hay or grass *ad libitum* until sacrifice. In this study, there were 37 pregnancies, of which 25 were twins and 20 where at least one twin was female. We studied only offspring from twin pregnancies and used one female offspring per pregnancy (C = 10; PA = 10).

Sheep were sacrificed by barbiturate overdose in accordance with the regulatory outline of Schedule 1: Appropriate Methods of Humane Killing of the Animals (Scientific Procedures) Act 1986. Offspring from the 22-month-old cohort were killed when the ovaries were seasonally quiescent to minimise differences in reproductive hormones. SAT from the groin region was recovered for histological analysis (Bouin’s solution fixation for 24 h, then transferred to 70% ethanol before processing and embedding in paraffin wax) and mRNA-based analyses (snap-frozen and stored at −80°C until further processing).

### Adipocyte morphometric analysis

Fixed SAT was investigated for adipocyte morphometric analysis. Six 5 μm sections were cut per adipose tissue sample, a minimum of 100 μm apart, and mounted on positively charged slides (Epredia, USA). Sections were then stained with haematoxylin and eosin following standard protocols. Two randomly selected fields per section were captured at 20× magnification using a Zeiss microscope (ZEISS Axioscan Z1 slide scanner; Carl Zeiss, Germany). Images were analysed using ImageJ software in a blinded manner. Data were averaged for each individual sheep.

### RNA extraction

RNA extraction from frozen adipose tissue (*n* = 20; C = 10, PA = 10) was performed using the TRI reagent combined with the RNeasy Lipid Tissue Mini Kit (Cirera 2013, Roy *et al.* 2020) (QIAGEN Ltd, UK) as per the manufacturer’s instructions. Adipose tissue (100 mg) samples were homogenised in 600 μL TRI reagent using a Qiagen TissueLyser (4 min, 30 Hz) and incubated at room temperature (RT) for 5 min. Chloroform (200 μL) was added, and the samples were shaken vigorously for 15 s. Samples were incubated for 3 min at RT and centrifuged (12,000 *g* at 4°C) for 30 min. The upper aqueous phase containing RNA was transferred into new Eppendorf tubes and then mixed 1:1 with 70% ethanol. After this step, RNA was extracted following the manufacturer’s instructions and stored at −80°C. RNA concentration and purity were measured using a NanoDrop 1000 spectrophotometer (Thermo Fisher Scientific, UK). RNA integrity number (RIN) scores, generated using LabChip GX (Software Version 5.10.172.0), ≥7 were used as a threshold for downstream analysis.

### Quantitative real-time (qRT)-PCR

Complementary DNA (cDNA) was synthesised (Precision Reverse Transcription Premix Kit (Primer Design, UK)) using 400 ng total RNA per sample. We previously described suitable, stable housekeeping genes for ovine SAT (Siemienowicz *et al.* 2019), and in this study, we used *ACTB*. Quantitative RT-PCR was performed using the ABI 7900HT Fast system (Applied Biosystems, UK). Primers were designed and validated as previously described (Siemienowicz *et al.* 2021) (Supplementary Table S1 (see section on Supplementary materials given at the end of the article)). Negative controls consisted of no-template and RT-negative controls. The expression of the target gene relative to the housekeeping gene was quantified using the ^2−^ΔΔCt method.

### Library creation and sequencing

Libraries were prepared with the Illumina TruSeq Stranded mRNA kit using BMAT control (*n* = 10) and PA samples (*n* = 10). Sequencing was performed on the NovaSeq 150 PE platform (Illumina, UK). Reads were trimmed using Cutadapt (Kechin *et al.* 2017) (version cutadapt-1.18-venv) using a quality threshold of 30. Reads were aligned to the reference genome (*Ovis aries* version GRCh38 downloaded from Ensembl) using STAR (Dobin *et al.* 2013) (version 2.7.3a) with paired-end reads. Reads were counted at gene locations using featureCounts (Liao *et al.* 2014) (version 1.5.1). In addition to the counts matrix used in downstream differential analysis, a matrix of fragments per kilobase of transcript per million mapped reads (FPKM) values was generated using the rpkm function of edgeR (Robinson *et al.* 2009) (version 3.28.1) and normalised effective library sizes. The raw counts table was filtered to remove genes consisting predominantly of near-zero counts, filtering on counts per million (CPM) to avoid artefacts due to library depth, leaving 15,539 genes for analysis. Reads were normalised using the weighted trimmed mean of M-values method (Robinson & Oshlack 2010), passing ‘TMM’ as the method to the calcNormFactors method of edgeR. A principal components analysis was undertaken on normalised and filtered expression data to explore observed patterns with respect to experimental factors. The cumulative proportion of variance associated with each component was used to study the level of structure in the data. Associations between principal components and categorical experimental factors were assessed with an ANOVA test, and associations with numerical variables were assessed using the Pearson correlation.

Pairwise gene comparisons were carried out with edgeR (version 3.28.1). *Q* values set at *q* < 0.05 using the Benjamini–Hochberg method were used to inform the false discovery rate. Library creation and sequencing were conducted by Edinburgh Genomics.

### Bioinformatics analysis process

Differential gene set analysis was carried out with the CAMERA method (Wu & Smyth 2012) from the Limma package (Ritchie *et al.* 2015) (version 3.42.2) of Bioconductor, using the same models and contrasts as used in differential expression. Each gene set was annotated with those genes individually differentially expressed (in the same direction as indicated for the gene set) to an unadjusted *P*-value of 0.05. In addition, an Ingenuity Pathway Analysis (IPA) (Qiagen, UK) was performed to further explore the biological pathways and networks related to the differentially expressed genes (DEGs). Pre-filtering of data uploaded to IPA was based on nominal *P* < 0.05 as a cut-off. IPA *P*-values of <0.05, calculated with right-tailed Fisher’s exact test, provided information on potential links of significant DEGs with biological pathways or diseases, and absolute Z-scores ≥2/−2 delivered information on directional change relating to such biological pathways.

### Masson trichrome staining

Masson trichrome staining on 5 μm adipose tissue sections was performed using the Trichrome Stain Kit (Abcam, UK) following the manufacturer’s protocol (*n* = 3 per animal, at least 100 μm apart). A Zeiss microscope (ZEISS Axioscan Z1 slide scanner; Carl Zeiss, Germany) was used to acquire the images. The percentage of fibrosis area (μm^2^) per animal (collagen stained blue with Masson trichrome) was measured in a blinded manner using ImageJ software, applying a consistent threshold (Baidoo *et al.* 2023).

### Intravenous glucose tolerance testing

Sheep were fasted overnight, and a basal blood sample was taken and decanted into a heparinised tube for later plasma insulin assessment and into a tube containing sodium fluoride for blood glucose measurement. After an intravenous bolus of glucose (10 g in 20 mL saline), repeat blood samples were taken at 15, 30 and 45 min. Tubes were centrifuged at 1,200 *g* for 15 min at 4°C, and plasma was collected and stored at −20°C. Glucose was measured with an enzymatic-colourimetric assay kit (Alpha Laboratories Ltd, UK) using a Cobas Fara analyser (Roche Diagnostics Ltd, UK). Assay sensitivity was 0.2 mmol/L, and the intra- and inter-assay CVs were <2% and <3%, respectively. Insulin was measured using the ALPCO Ovine Insulin ELISA kit (American Laboratory Products Company, USA) following the manufacturer’s instructions. The assay sensitivity was 0.14 ng/mL, and the intra- and inter-assay CVs were <5% and <6%, respectively.

### Statistical analysis

Data analyses were performed using the Statistical Package for the Social Sciences (SPSS 17.0 for windows; IBM, USA). Between-group differences were analysed by *t*-test, corrected *t*-test (according to whether the homogeneity of variance was satisfied) or Mann–Whitney U test owing to the non-normality of data. *P* ≤ 0.05 was considered statistically significant. Figures were prepared using GraphPad Prism version 8.0 (GraphPad software, Inc., USA).

## Results

### SAT morphology in PCOS-like young adult sheep

**Figure 1:**
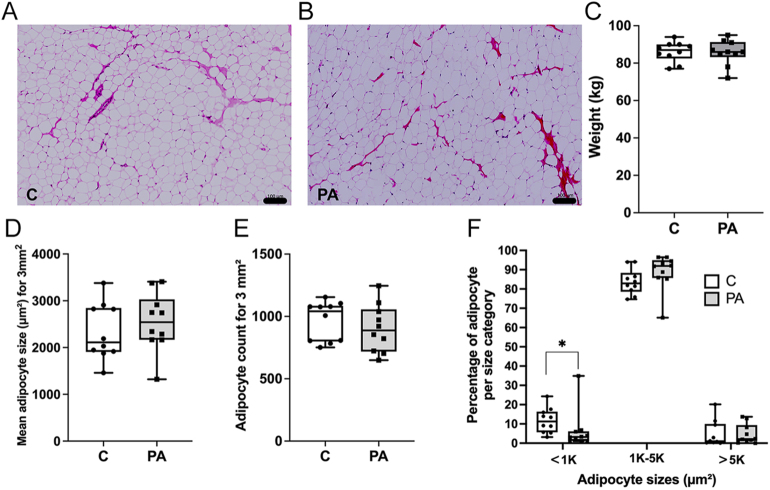
Subcutaneous adipose morphology analysis in young adults. Histological analysis of SAT morphology in control (C; *n* = 10) (A) and PCOS-like sheep (PA; *n* = 10) (B) that are weight matched (C). In SAT, there was no difference in the mean size of adipocytes (D) and no difference in the numbers of adipocytes per mm^2^ (E). There was a slightly reduced number of small adipocytes but no alteration in the number of adipocytes of medium and large sizes (F). (Scale bars = 100 μm). Data were analysed by *t*-test and Mann–Whitney test where variances were different. *P < 0.05. Box plot whiskers are lowest and highest observed values; box is the upper and lower quartile, with median represented by the line in the box.

SAT morphology (Fig. 1A and B) was compared in weight-matched (Fig. 1C) PCOS-like (*n* = 10) and control (*n* = 10) female sheep in young adulthood (22 months old). Morphometric analysis showed no difference in mean adipocyte size (Fig. 1D) or number of adipocytes per mm^2^ (Fig. 1E). However, detailed analysis showed that, while there were no differences in medium-sized (1,000–5,000 μm^2^) or large-sized (>5,000 μm^2^) adipocytes, there was a reduction (*P* < 0.05) in small-sized (<1,000 μm^2^) adipocytes in PCOS-like sheep when compared to controls (Fig. 1F). Thus, at 22 months of age, SAT structure in PCOS-like sheep is similar to that in control sheep.

### Adipogenesis and inflammation in SAT in young adult sheep

**Figure 2:**
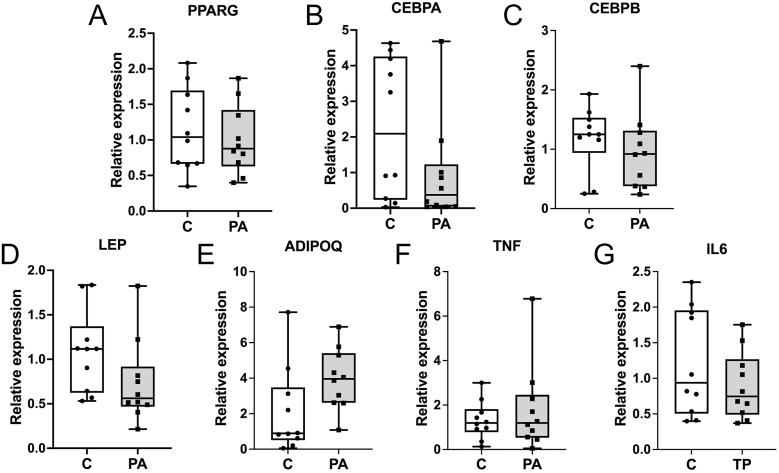
Expression of adipogenic, functional and inflammatory markers in SAT. There was no significant difference in the expression of the adipogenesis markers *PPARG* (A), *CEBPA* (B) and *CEBPB* (C) between control (C; *n* = 10) and PCOS-like (PA; *n* = 10) young adult sheep. The functional markers *LEP* (D) and *ADIPOQ* (E) were also not differentially expressed. In addition, the expression of the inflammatory markers *TNF* (F) and *IL6* (G) was not different. Data were analysed by *t*-test and by Mann–Whitney test where variances were different.

At 22 months of age, PCOS-like sheep do not have altered expression of key adipogenesis-related genes, as assessed by *PPARG* (Fig. 2A), *CEBPA* (Fig. 2B) and *CEBPB* (Fig. 2C) transcript abundance. In addition, there was no difference in adipocyte *LEP* (Fig. 2D) or *ADIPOQ* (Fig. 2E) expression. There was no evidence of increased inflammation, assessed by *TNF* (Fig. 2F) and *IL6* (Fig. 2G) transcript abundance, in PCOS-like sheep when compared to controls. At 22 months, pathways that are dysregulated in adolescence and adulthood in PCOS-like sheep are normal.

### Analysis of SAT gene expression in young adult sheep through non-biased RNAseq

**Figure 3:**
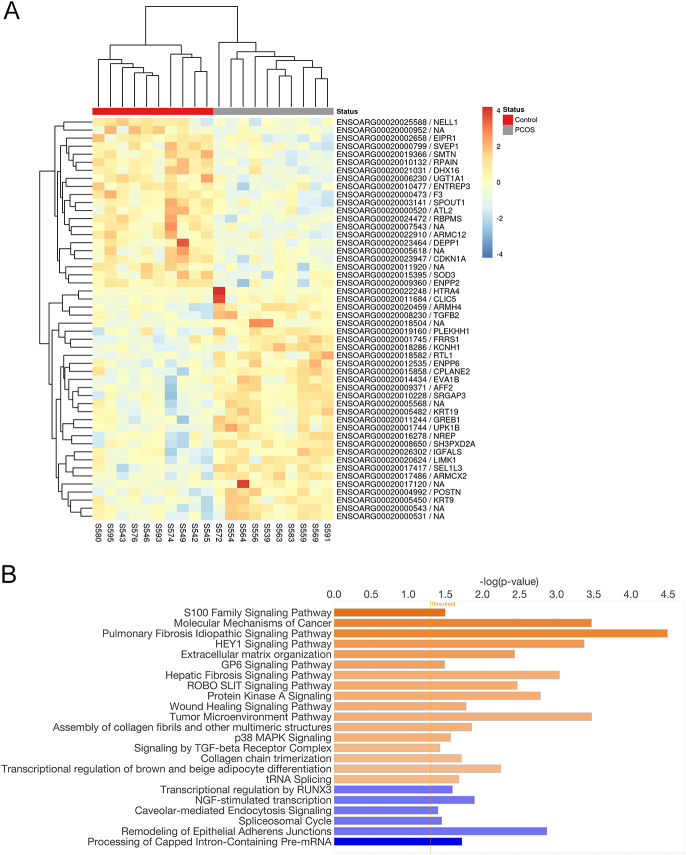
Differentially expressed transcripts analysis. (A) Heatmap of the top 50 most variant rows of the normalised expression matrix. Figure generated with the ‘heatmap’ package. (B) Results of IPA highlighting the most altered pathways between control and PCOS-like sheep. From Qiagen IPA analysis, all rights reserved.

To examine early markers of future SAT dysfunction, RNAseq analysis was performed. The mRNA libraries generated a total of 19.6–51.3 million read pairs and 18.8–48.7 million read pairs after trimming (94.2–95.8%). Between 53.6 and 58.3% of the clean read pairs were mapped and assigned to features for counting, and 25,916 genes were counted before filtering, of which 15,539 remained after filtering. A total of 792 genes were identified as significantly differentially expressed (*P* < 0.05) in SAT between the PA sheep and the controls, including 406 upregulated and 386 downregulated, although none passed the robust threshold of *q* < 0.05. The top 50 most variant genes are listed in a heatmap of expression values (Fig. 3A). Consistent with qPCR analysis, *PPARG, CEBPA, CEBPB, LEP, ADIPOQ*, *TNF* and *IL6* transcripts did not appear among the DEGs. IPA (Fig. 3B) highlighted upregulation of fibrosis signalling pathways, extracellular organisation pathways, and the assembly of collagen fibrils. Fibrotic pathways are upregulated in the SAT of young adult PCOS-like sheep when compared to controls.

### Structural and functional analysis of SAT fibrosis

**Figure 4:**
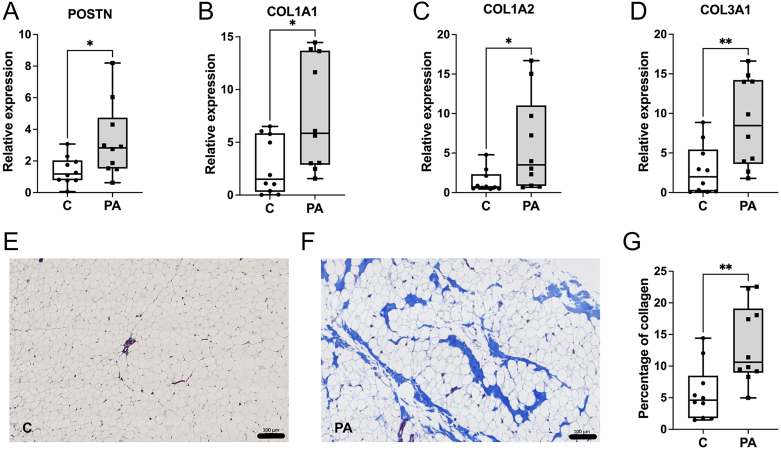
Collagen gene expression and Masson’s trichrome staining in SAT. The expression levels of *POSTN* (A), *COLA1A* (B), *COL1A2* (C) and *COL3A1* (D) were increased in the SAT of PCOS-like (PA; *n* = 10) sheep when compared to control sheep (C; *n* = 10). Masson’s trichrome staining of SAT in (E) control sheep and (F) PCOS-like sheep. There was increased collagen histochemical staining in PCOS-like sheep SAT when compared to control sheep SAT (G). (Scale bars = 100 μm). Data were analysed by *t*-test and Mann–Whitney test where variances were different; *P < 0.05, **P < 0.01. Box plot whiskers are lowest and highest observed values; box is the upper and lower quartile, with median represented by the line in the box.

**Figure 5:**
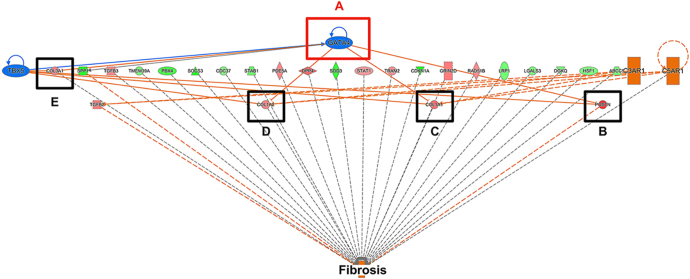
Regulatory interaction of collagen gene expression. IPA analysis highlighted that GATA4 transcription factor (A) activation was commonly associated with the regulation of *POSTN* (B), *COLA1A* (C), *COL1A2* (D) and *COL3A1* (E). Qiagen, all rights reserved.

Functionally, *POSTN*, which is associated with response to injury and tissue fibrosis, was increased (Fig. 4A; *P* < 0.05) in PCOS-like sheep. In addition, the expression of collagen gene transcripts *COL1A1* (Fig. 4B; *P* < 0.05), *COL1A2* (Fig. 4C; *P* < 0.05) and *COL3A1* (Fig. 4D; *P* < 0.05) was increased. Histochemical assessment of fibrosis in SAT was examined using Masson’s trichrome staining of collagen. In the control sheep (Fig. 4E), minimal SAT fibrosis was found, with a mean value of 5.75% ± 1.39% (Fig. 4G). However, in the PCOS-like sheep (Fig. 4F), a significant increase in the amount of collagen staining was observed, with a mean value of 13.34% ± 1.96% (Fig. 4G; *P* < 0.05). Pathway analysis revealed a common regulatory link with *GATA4* transcription factor activity (Fig. 5).

### The evolution of insulin resistance

**Figure 6:**
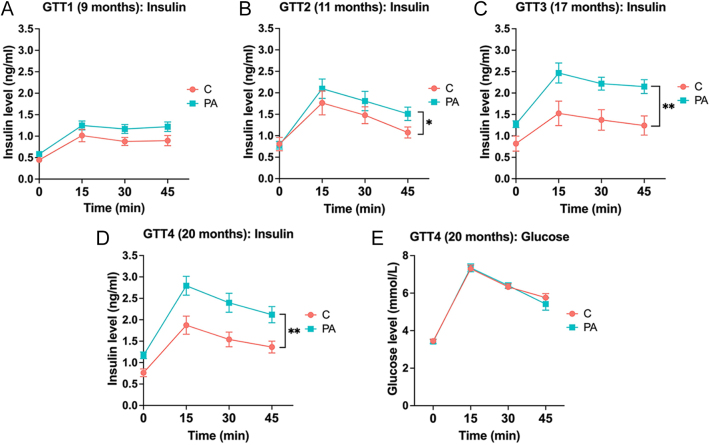
The evolution of IR. Serum insulin levels in the 45 min after an intravenous GTT in control sheep (*n* = 10) and PCOS-like (PA) sheep at 9 months of age (A), 11 months of age (B), 17 months of age (C) and 22 months of age (D). Serum glucose levels in the 45 min after GTT at 22 months of age (E). Data were analysed by AUC assessment, and where significant differences existed, time differences were assessed by *t*-tests; *P < 0.05, **P < 0.005.

As GATA4 function can be regulated by insulin, we examined the evolution of IR in these sheep. At 9 months, there were no significant differences in plasma insulin area under the curve (AUC) concentrations during an intravenous glucose tolerance test (GTT) in PCOS-like sheep when compared to controls (Fig. 6A). However, PCOS-like sheep had increased AUC insulin concentrations during adolescence at 11 months of age (Fig. 6B (*P* < 0.05)), which persisted into young adulthood (Fig. 6C and D; *P* < 0.005). There were no differences in the serum glucose concentrations during a GTT between the two groups (Fig. 6E).

## Discussion

In this study, we examined the evolution of adipocyte dysfunction in PCOS by using a clinically realistic sheep model at 22 months of age (young adults). To maximise the availability of disparate tissues, we studied different cohorts of sheep at different time points rather than one cohort longitudinally. We have reported decreased SAT adipogenesis in adolescence (11 months) that predisposes to decreased lipid storage capacity in adulthood (30 months), with adipocyte hypertrophy and markers of inflammation (Siemienowicz *et al.* 2021). In mature adulthood, there is also established IR, increased thecal androgen synthesis and anovulation (Hogg *et al.* 2012, Siemienowicz *et al.* 2020).

Our results show that this young adult stage, after the post-pubertal adipogenesis wave is largely complete but before inflammatory transcriptome markers are increased, had no attendant alterations in adipocyte size or differences in the expression of inflammatory (*TNF* and *IL6*) and adipogenesis (*PPARG*, *CEBPA* and *CEBPB*) markers. However, RNAseq analysis indicated upregulation of fibrosis signalling pathways in PA sheep. This suggestion was further cemented via qPCR, demonstrating significantly increased expression of *POSTN*, *COL1A1, COL1A2* and *COL3A*. These gene expression markers of increased fibrotic potential were confirmed via histological analysis, demonstrating a significant increase in the amount of collagen deposition in the SAT of PCOS-like sheep. In addition, there were increased insulin concentrations in the PA sheep during adolescence, which persisted into young adulthood. IPA analysis highlighted the transcription factor *GATA4*, a fibrosis regulator, upstream of the fibrotic gene expression changes underpinning increased fibrotic deposition, which is known to be regulated by insulin, and we suggest it may be involved in fibrotic pathway increases.

PCOS is linked with functional adipose tissue alteration; however, the mechanisms underlying this association remain incompletely understood, both in terms of mechanism and chronology. The structure of SAT, including adipocyte size and the content ratio of different-sized adipocytes, is crucial for normal metabolic function (Laforest *et al.* 2015, Tandon *et al.* 2018, Ghaben & Scherer 2019). In adults with obesity, the adipocytes in SAT are enlarged, resulting in a relatively low capillary density and local hypoxia, with an accompanying increase in local inflammation and fibrosis (Ahn *et al.* 2024). We reported previously that the structure of SAT was altered, with larger adipocytes and less capacity for thermogenesis, in the older adult (30 months) PCOS-like female sheep (Siemienowicz *et al.* 2021), which is in agreement with findings from human PCOS studies (Mannerås-Holm *et al.* 2011, Echiburú *et al.* 2018, Bril *et al.* 2023, Abbasi *et al.* 2024). The present study demonstrates that there was no difference in the average size and number of SAT adipocytes between the weight-matched cohort of 22-month-old PA and control sheep.

During adipogenesis, the terminal differentiation of pre-adipocytes to mature adipocytes involves increasing capacity for lipid transport, synthesis and storage, insulin responsiveness and adipokine synthesis. Adipogenesis develops as a two-step process: undifferentiated mesenchymal cells convert into pre-adipocytes, which then differentiate into lipid-filled adipocytes (Yang *et al.* 2023). Peroxisome proliferator-activated receptor (PPAR-ɣ) and the CCAAT/enhancer-binding protein transcription factor family (*C/EBP*) are involved in the regulation of the adipogenesis process (Mota de Sá *et al.* 2017). CEBPA and PPARG induce and maintain the expression of key adipogenic genes, including adiponectin (*ADIPOQ*), which are necessary for normal adipocyte function. We have found that altered adipogenesis in SAT occurs during adolescence in PCOS-like sheep (11 months) (Siemienowicz *et al.* 2021). However, key transcriptional regulators of adipogenesis, *PPARG*, *CEBPA*, *CEBPB* and *ADIPOQ* were not altered in young adult SAT. In addition, the inflammatory marker tumour necrosis factor (*TNF*) and interleukin 6 (*IL6*) transcripts, which were found to be increased in SAT in adult PCOS-like sheep, were not changed in the young adult sheep. This suggests that the detrimental inflammatory environment in SAT in PCOS may not have developed at this stage and that this time point could be used to identify novel key pathways underpinning later SAT dysfunction that is linked to metabolic disturbances (Goodarzi *et al.* 2011, Sanchez-Garrido & Tena-Sempere 2020, Bril *et al.* 2023).

By comparing the SAT of PA sheep with the control group, we found that overall gene expression was similar when accounting for FDR. However, we identified a total of 792 potentially DEGs (*P* < 0.05), including 406 upregulated and 386 downregulated genes (Supplementary Table S2). To analyse the links between potential DEGs, IPA analysis was used. This indicated upregulation of multiple pathways related to collagen production and fibrosis, including, ‘collagen chain trimerization’, ‘wound healing signalling pathway’ and ‘assembly of collagen fibrils and other multimeric structures’. These findings suggest that fibrosis contributes to the development of PCOS-related SAT dysfunction.

A gradual and healthy expansion of adipose tissue in response to caloric intake is associated with appropriate vascular and extracellular matrix (ECM) remodelling (Halberg *et al.* 2009). Healthy SAT expansion is generally associated with the presence of smaller and more numerous adipocytes. SAT fibrosis is a characteristic feature of obesity and lipodystrophy, and it is associated with inflammation and IR (Householder *et al.* 2017). In addition, SAT fibrosis negatively impacts metabolism by limiting healthy adipocyte expansion. Collagen production by adipocytes heavily influences adipose tissue expandability. Abnormal collagen deposition is a key feature of adipose tissue dysfunction (Sun *et al.* 2013).

Our study suggests that collagen production was upregulated. This is evidenced by significant increases in the expression of pro-fibrogenic target genes (*COL1A1*, *COL1A2* and *COL3A1*) and collagen deposition. IPA also identified periostin (*POSTN*) as another key gene linked to fibrosis pathways. *POSTN* has already been identified as a novel regulator of adipose tissue fibrosis (Nakazeki *et al.* 2018). Its expression was found to be significantly increased in mouse adipose tissue after treatment with lipopolysaccharide or a high-fat diet, and adipose progenitor cells are the main source of POSTN (Yang *et al.* 2021). In the case of metabolic disease, there is experimental evidence implicating POSTN in hepatic steatosis, inflammation, and fibrosis (Yan *et al.* 2014).

The association between SAT fibrosis and dysfunction in PCOS is not fully understood. Like other fibrotic diseases, adipose tissue fibrosis involves the accumulation and increased production of ECM proteins (Buechler *et al.* 2015). Collagen gene expression is tightly regulated by various factors, including transforming growth factor beta (TGF-β) (Nigdelioglu *et al.* 2016), lysyl oxidase (LOX) (Herchenhan *et al.* 2015), hypoxia (Gilkes *et al.* 2014), and inflammation (Narayanan *et al.* 1983), all of which contribute to the overall fibrotic process. In addition, a series of genes involved in the maintenance and regulation of the ECM were significantly changed in PCOS-like sheep SAT (Supplementary Table S3).

Bioinformatic analysis showed that the four highlighted genes (*COL1A1*, *COL1A2*, *COL3A1* and *POSTN*) were linked to the transcription factor GATA binding protein 4 (*GATA4*). The loss of intestinal GATA4 prevents diet-induced obesity, promotes insulin sensitivity, and protects against diet-induced hepatic steatosis in mice (Patankar *et al.* 2012). Although in the current work *GATA4* was found not to be significantly changed in the RNAseq results, there is a post-transcriptional mode of regulation of GATA4 expression. A study showed that hypertrophic mouse hearts had a significant increase in GATA4 protein levels without a corresponding increase in *GATA4* mRNA (Han *et al.* 2012). Its function can also be regulated by phosphorylation (Liang *et al.* 2001), and sourcing good phospho-GATA4 antibodies that work in ovine tissues is challenging. Moreover, it was discovered that GATA4 could affect members of insulin signalling, including IFG-1 (Bennett *et al.* 2013), IGFBP4 (Bennett *et al.* 2013) and IGFBP5 (Li *et al.* 2020), all of which were found significantly changed in PA SAT (Supplementary Fig. 1). Given the relationship of GATA4 with insulin sensitivity, and to further verify our results, we examined the evolution of IR in these sheep. Insulin levels were increasingly higher with age in PA sheep when compared to controls. However, there were no differences in the serum glucose concentrations between the two groups.

There are limitations to our study. The first is that the effects in adolescence and adulthood we quote were in different cohorts of sheep using the same reproducible treatment regimen. This is not a longitudinal study from birth to adulthood with multiple SAT biopsies. The second is that we report a lack of differences in adipogenesis genes and inflammatory genes only at the gene level and not at the protein level within the SAT tissue. In addition, we used quantification of histochemical staining of collagen as a marker of fibrosis rather than measurement of specific collagen proteins using antibody methods. The link between GATA4, SAT fibrosis and IR is only by association and speculative, and more research is needed to examine whether GATA4 is the master regulator of fibrotic changes in SAT and if its function is related to insulin sensitivity.

In summary, we have shown that the PA sheep had significant SAT fibrosis in view of molecular and histopathological changes. This indicates that fibrosis may contribute to the development of adipose tissue dysfunction in PCOS, and GATA4 might be a potential regulator. The early fibrotic changes in SAT, at this developmental stage of 22 months, occur before changes in adipocyte size and inflammatory transcription markers in these PCOS-like sheep. This might provide a barrier to healthy adipocyte expansion and have a mechanistic role in the development of the detrimental inflammatory environment in PCOS. There may be a therapeutic window before young adulthood that can allow targeting of adipose tissue fibrosis to improve metabolic health in PCOS.

## Supplementary materials









## Declaration of interest

The authors declare that there is no conflict of interest that could be perceived as prejudicing the impartiality of the work reported.

## Funding

This work was funded by Medical Research Councilhttps://doi.org/10.13039/501100000265 (MRC) project grants (G0801807 and MR/W015439/1) and an academic scholarship from the Society for Reproduction and Fertility (SRF).

## References

[bib1] Abbasi K, Zarezadeh R, Valizadeh A, et al. 2024 White-brown adipose tissue interplay in polycystic ovary syndrome: therapeutic avenues. Biochem Pharmacol 220 116012. (10.1016/j.bcp.2023.116012) 38159686

[bib2] Ahn C, Zhang T, Yang G, et al. 2024 Years of endurance exercise training remodel abdominal subcutaneous adipose tissue in adults with overweight or obesity. Nat Metab 6 1819–1836. (10.1038/s42255-024-01103-x) 39256590 PMC13157827

[bib3] Baidoo N, Sanger GJ & Belai A 2023 Histochemical and biochemical analysis of collagen content in formalin-fixed, paraffin embedded colonic samples. MethodsX 11 102416. (10.1016/j.mex.2023.102416) 37876831 PMC10590991

[bib4] Balen A 2004 The pathophysiology of polycystic ovary syndrome: trying to understand PCOS and its endocrinology. Best Pract Res Clin Obstet Gynaecol 18 685–706. (10.1016/j.bpobgyn.2004.05.004) 15380141

[bib5] Bennett J, Baumgarten SC & Stocco C 2013 GATA4 and GATA6 silencing in ovarian granulosa cells affects levels of mRNAs involved in steroidogenesis, extracellular structure organization, IGF-I activity, and apoptosis. Endocrinology 154 4845–4858. (10.1210/en.2013-1410) 24064357 PMC3836082

[bib6] Birch RA, Padmanabhan V, Foster DL, et al. 2003 Prenatal programming of reproductive neuroendocrine function: fetal androgen exposure produces progressive disruption of reproductive cycles in sheep. Endocrinology 144 1426–1434. (10.1210/en.2002-220965) 12639926

[bib7] Bril F, Ezeh U, Amiri M, et al. 2023 Adipose tissue dysfunction in polycystic ovary syndrome. J Clin Endocrinol Metab 109 10–24. (10.1210/clinem/dgad356) 37329216 PMC10735305

[bib8] Buechler C, Krautbauer S & Eisinger K 2015 Adipose tissue fibrosis. World J Diabetes 6 548–553. (10.4239/wjd.v6.i4.548) 25987952 PMC4434075

[bib9] Cirera S 2013 Highly efficient method for isolation of total RNA from adipose tissue. BMC Res Notes 6 472. (10.1186/1756-0500-6-472) 24245791 PMC4225616

[bib10] de Medeiros SF, Rodgers RJ & Norman RJ 2021 Adipocyte and steroidogenic cell cross-talk in polycystic ovary syndrome. Hum Reprod Update 27 771–796. (10.1093/humupd/dmab004) 33764457

[bib11] Dobin A, Davis CA, Schlesinger F, et al. 2013 STAR: ultrafast universal RNA-seq aligner. Bioinformatics 29 15–21. (10.1093/bioinformatics/bts635) 23104886 PMC3530905

[bib12] Echiburú B, Pérez-Bravo F, Galgani JE, et al. 2018 Enlarged adipocytes in subcutaneous adipose tissue associated to hyperandrogenism and visceral adipose tissue volume in women with polycystic ovary syndrome. Steroids 130 15–21. (10.1016/j.steroids.2017.12.009) 29273198

[bib13] Fauser BCJM, Tarlatzis BC, Rebar RW, et al. 2012 Consensus on women’s health aspects of polycystic ovary syndrome (PCOS): the Amsterdam ESHRE/ASRM-sponsored 3rd PCOS consensus workshop group. Fertil Steril 97 28–38. (10.1016/j.fertnstert.2011.09.024) 22153789

[bib14] Ghaben AL & Scherer PE 2019 Adipogenesis and metabolic health. Nat Rev Mol Cell Biol 20 242–258. (10.1038/s41580-018-0093-z) 30610207

[bib15] Gilkes DM, Semenza GL & Wirtz D 2014 Hypoxia and the extracellular matrix: drivers of tumour metastasis. Nat Rev Cancer 14 430–439. (10.1038/nrc3726) 24827502 PMC4283800

[bib16] Goodarzi MO, Dumesic DA, Chazenbalk G, et al. 2011 Polycystic ovary syndrome: etiology, pathogenesis and diagnosis. Nat Rev Endocrinol 7 219–231. (10.1038/nrendo.2010.217) 21263450

[bib17] Halberg N, Khan T, Trujillo ME, et al. 2009 Hypoxia-inducible factor 1α induces fibrosis and insulin resistance in white adipose tissue. Mol Cell Biol 29 4467–4483. (10.1128/mcb.00192-09) 19546236 PMC2725728

[bib18] Han M, Yang Z, Sayed D, et al. 2012 GATA4 expression is primarily regulated via a miR-26b-dependent post-transcriptional mechanism during cardiac hypertrophy. Cardiovasc Res 93 645–654. (10.1093/cvr/cvs001) 22219180 PMC3291090

[bib19] Herchenhan A, Uhlenbrock F, Eliasson P, et al. 2015 Lysyl oxidase activity is required for ordered collagen fibrillogenesis by tendon cells. J Biol Chem 290 16440–16450. (10.1074/jbc.m115.641670) 25979340 PMC4481240

[bib20] Hoeger KM, Dokras A & Piltonen T 2021 Update on PCOS: consequences, challenges, and guiding treatment. J Clin Endocrinol Metab 106 e1071–e1083. (10.1210/clinem/dgaa839) 33211867

[bib21] Hogg K, Young JM, Oliver EM, et al. 2012 Enhanced thecal androgen production is prenatally programmed in an ovine model of polycystic ovary syndrome. Endocrinology 153 450–461. (10.1210/en.2011-1607) 22087026

[bib22] Householder LA, Comisford R, Duran-Ortiz S, et al. 2017 Increased fibrosis: a novel means by which GH influences white adipose tissue function. Growth Horm IGF Res 39 45–53. (10.1016/j.ghir.2017.12.010) 29279183 PMC5858978

[bib23] Kechin A, Boyarskikh U, Kel A, et al. 2017 cutPrimers: a new tool for accurate cutting of primers from reads of targeted next generation sequencing. J Comput Biol 24 1138–1143. (10.1089/cmb.2017.0096) 28715235

[bib24] Laforest S, Labrecque J, Michaud A, et al. 2015 Adipocyte size as a determinant of metabolic disease and adipose tissue dysfunction. Crit Rev Clin Lab Sci 52 301–313. (10.3109/10408363.2015.1041582) 26292076

[bib25] Levate G, Wang Y, McCredie R, et al. 2024 Insights into the effects of sex and tissue location on the evolution of adipocyte dysfunction in an ovine model of polycystic ovary syndrome (PCOS). Mol Cell Endocrinol 595 112416. (10.1016/j.mce.2024.112416) 39557184

[bib26] Li X, Zhou Q, Wang S, et al. 2020 Prolonged treatment with Y-27632 promotes the senescence of primary human dermal fibroblasts by increasing the expression of IGFBP-5 and transforming them into a CAF-like phenotype. Aging 12 16621–16646. (10.18632/aging.103910) 32843583 PMC7485707

[bib27] Liang Q, Wiese RJ, Bueno OF, et al. 2001 The transcription factor GATA4 is activated by extracellular signal-regulated kinase 1- and 2-mediated phosphorylation of serine 105 in cardiomyocytes. Mol Cell Biol 21 7460–7469. (10.1128/mcb.21.21.7460-7469.2001) 11585926 PMC99918

[bib28] Liao Y, Smyth GK & Shi W 2014 featureCounts: an efficient general purpose program for assigning sequence reads to genomic features. Bioinformatics 30 923–930. (10.1093/bioinformatics/btt656) 24227677

[bib29] Mannerås-Holm L, Leonhardt H, Kullberg J, et al. 2011 Adipose tissue has aberrant morphology and function in PCOS: enlarged adipocytes and low serum adiponectin, but not circulating sex steroids, are strongly associated with insulin resistance. J Clin Endocrinol Metab 96 E304–E311. (10.1210/jc.2010-1290) 21084397

[bib30] Mota de Sá P, Richard AJ, Hang H, et al. 2017 Transcriptional regulation of adipogenesis. Compr Physiol 7 635–674. (10.1002/j.2040-4603.2017.tb00753.x) 28333384

[bib31] Nakazeki F, Nishiga M, Horie T, et al. 2018 Loss of periostin ameliorates adipose tissue inflammation and fibrosis in vivo. Sci Rep 8 8553. (10.1038/s41598-018-27009-9) 29867212 PMC5986813

[bib32] Narayanan AS, Engel LD & Page RC 1983 The effect of chronic inflammation on the composition of collagen types in human connective tissue. Collagen Relat Res 3 323–334. (10.1016/s0174-173x(83)80014-4) 6413124

[bib33] Nigdelioglu R, Hamanaka RB, Meliton AY, et al. 2016 Transforming growth factor (TGF)-β promotes de novo serine synthesis for collagen production. J Biol Chem 291 27239–27251. (10.1074/jbc.m116.756247) 27836973 PMC5207151

[bib34] Patankar JV, Obrowsky S, Doddapattar P, et al. 2012 Intestinal GATA4 deficiency protects from diet-induced hepatic steatosis. J Hepatol 5 1061–1068. (10.1016/j.jhep.2012.06.028) PMC3477492 22750465

[bib35] Ramaswamy S, Grace C, Mattei AA, et al. 2016 Developmental programming of polycystic ovary syndrome (PCOS): prenatal androgens establish pancreatic islet α/β cell ratio and subsequent insulin secretion. Sci Rep 6 27408. (10.1038/srep27408) 27265420 PMC4893678

[bib36] Ritchie ME, Phipson B, Wu D, et al. 2015 Limma powers differential expression analyses for RNA-sequencing and microarray studies. Nucleic Acids Res 43 e47. (10.1093/nar/gkv007) 25605792 PMC4402510

[bib37] Robinson MD & Oshlack A 2010 A scaling normalization method for differential expression analysis of RNA-seq data. Genome Biol 11 220. (10.1186/gb-2010-11-3-r25) 20196867 PMC2864565

[bib38] Robinson MD, McCarthy DJ & Smyth GK 2009 edgeR: a bioconductor package for differential expression analysis of digital gene expression data. Bioinformatics 26 139–140. (10.1093/bioinformatics/btp616) 19910308 PMC2796818

[bib39] Roy D, Tomo S, Modi A, et al. 2020 Optimising total RNA quality and quantity by phenol-chloroform extraction method from human visceral adipose tissue: a standardisation study. MethodsX 7 101113. (10.1016/j.mex.2020.101113) 33204654 PMC7653057

[bib40] Sanchez-Garrido MA & Tena-Sempere M 2020 Metabolic dysfunction in polycystic ovary syndrome: pathogenic role of androgen excess and potential therapeutic strategies. Mol Metab 35 100937. (10.1016/j.molmet.2020.01.001) 32244180 PMC7115104

[bib41] Siemienowicz KJ, Filis P, Shaw S, et al. 2019 Fetal androgen exposure is a determinant of adult male metabolic health. Sci Rep 9 20195. (10.1038/s41598-019-56790-4) 31882954 PMC6934666

[bib42] Siemienowicz K, Rae MT, Howells F, et al. 2020 Insights into manipulating postprandial energy expenditure to manage weight gain in polycystic ovary syndrome. iScience 23 101164. (10.1016/j.isci.2020.101164) 32464593 PMC7256642

[bib43] Siemienowicz KJ, Coukan F, Franks S, et al. 2021 Aberrant subcutaneous adipogenesis precedes adult metabolic dysfunction in an ovine model of polycystic ovary syndrome (PCOS). Mol Cell Endocrinol 519 111042. (10.1016/j.mce.2020.111042) 33010309

[bib44] Stener-Victorin E, Padmanabhan V, Walters KA, et al. 2020 Animal models to understand the etiology and pathophysiology of polycystic ovary syndrome. Endocr Rev 41 bnaa010. (10.1210/endrev/bnaa010) 32310267 PMC7279705

[bib45] Stener-Victorin E, Teede H, Norman RJ, et al. 2024 Polycystic ovary syndrome. Nat Rev Dis Primers 10 27. (10.1038/s41572-024-00511-3) 38637590

[bib46] Sun K, Tordjman J, Clément K, et al. 2013 Fibrosis and adipose tissue dysfunction. Cell Metab 18 470–477. (10.1016/j.cmet.2013.06.016) 23954640 PMC3795900

[bib47] Tandon P, Wafer R & Minchin JEN 2018 Adipose morphology and metabolic disease. J Exp Biol 221 164970. (10.1242/jeb.164970) 29514883

[bib48] Wehr E, Möller R, Horejsi R, et al. 2009 Subcutaneous adipose tissue topography and metabolic disturbances in polycystic ovary syndrome. Wien Klin Wochenschr 121 262–269. (10.1007/s00508-009-1162-2) 19562283

[bib49] Wu D & Smyth GK 2012 Camera: a competitive gene set test accounting for inter-gene correlation. Nucleic Acids Res 40 e133. (10.1093/nar/gks461) 22638577 PMC3458527

[bib50] Yan M, Gingras M-C, Dunlop EA, et al. 2014 The tumor suppressor folliculin regulates AMPK-dependent metabolic transformation. J Clin Investig 124 2640–2650. (10.1172/jci71749) 24762438 PMC4038567

[bib51] Yang Y, Zhang Y, Zhou X, et al. 2021 Periostin deficiency attenuates lipopolysaccharide- and obesity-induced adipose tissue fibrosis. FEBS Lett 595 2099–2112. (10.1002/1873-3468.14154) 34165806

[bib52] Yang M, Gao X, Hu C, et al. 2023 Bta-miR-484 targets SFRP1 and affects preadipocytes proliferation, differentiation, and apoptosis. Int J Mol Sci 24 12710. (10.3390/ijms241612710) 37628891 PMC10454478

