# Supplementary figures and images for: An ovine model shows that subcutaneous adipose tissue fibrosis occurs early in polycystic ovary syndrome (PCOS)
Source: J Mol Endocrinol. 2025 Nov 19;75(4):e250106. doi: 10.1530/JME-25-0106 (PMC12630375; doi:10.1530/JME-25-0106)

Supplementary Figure 1

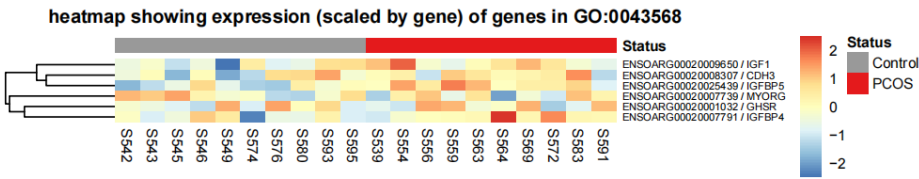

Supplement: Supplementary file 1 [file supplementary_figure.pdf]
