# Supplementary material for: An ovine model shows that subcutaneous adipose tissue fibrosis occurs early in polycystic ovary syndrome (PCOS)
Source: J Mol Endocrinol. 2025 Nov 19;75(4):e250106. doi: 10.1530/JME-25-0106 (PMC12630375; doi:10.1530/JME-25-0106)
Supplement: Supplementary file 2 [file supplementary_table_s1.pdf]

Supplementary Table S1

Primer information used for qRT-PCR

Gene	Forward sequence	Reverse sequence
PPARG	ACATTCCGTTCCCAAGAGC	GGGGATACAGGCTCCACTTT
CEBPA	GTGGACAAGAACAGCAACGA	CGCAGTGTGTCCAGTTCG
CEBPB	GCACAGCGACGAGTACAAGA	TATGCTGCGTCTCCAGGTTG
LEP	ATCTCACACACGCAGTCCGT	CCAGCAGGTGGAGAAGGTC
ADIPOQ	AGAGATGGCACCCCTGGT	GACCTTCGATCCCAGTGATT
TNF	GGTGCCTCAGCCTCTTCT	GAACCAGAGGCCTGTTGAAG
IL6	AAATGACACCACCCCAAGCA	CTCCAGAAGACCAGCAGTGG
POSTN	CCATCTGTGGACAGAAAACG	CACCATTTGTTGCAATCTGG
COL1A1	CGCTCCTTGTGTAACTGCAT	TTCACATGAGTCCCCATCCAC
COL1A2	GCCTATCCTTGATATTGCACCT	CTTTTGCCCACAATTTAAGCAAG
COL3A1	GAAAGCCTTGAAGCTGATGG	TGCTCTGAAAATGGGCTAGG
ACTB	ATCGAGGACAGGATGCAGAA	CCAATCCACACGGAGTACTTG
