# Supplementary material for: An ovine model shows that subcutaneous adipose tissue fibrosis occurs early in polycystic ovary syndrome (PCOS)
Source: J Mol Endocrinol. 2025 Nov 19;75(4):e250106. doi: 10.1530/JME-25-0106 (PMC12630375; doi:10.1530/JME-25-0106)
Supplement: Supplementary file 4 [file supplementary_table_s3.pdf]

Supplementary Table S3

Roles of collagen genes and their main regulators in fibrosis-related / adipose-related dysfunction

**Genes**	**Proteins**	**Results in RNAseq**	**Reported roles in fibrosis-related / adipose-related dysfunction**
**ECM components**			
COL1A1	Type I collagen	FC 1.55 P= 0.008	COL1A1 is significantly upregulated in various types of fibrosis, including idiopathic pulmonary fibrosis (IPF) (Tsitoura E et al. 2021) and liver and adipose tissue (Sun K et al. 2023) fibrosis, mainly regulated by TGF-β pathway (Devos H et al. 2023).<br>In heart failure (HF), upregulation of COL1A1 has been linked to the progression of fibrosis (Hua X et al. 2020).
COL1A2	Type I collagen	FC 1.35 P=0.014	COL1A2 expression is often elevated in various fibrotic conditions, including pulmonary fibrosis (Nassabeh N 2009) and cardiac (Pan X et al. 2022) fibrosis.<br>COL1A2 provides instructions for the production of type I collagen, leading to fibrotic conditions (Pan X et al. 2022).<br>Renal tubular epithelial cells exhibit increased COL1A2 transcription during the development of interstitial fibrosis in the kidneys (Fragiadaki M et al. 2011).
COL3A1	Type III collagen	FC 1.36 P=0.007	Overexpression of COL3A1, the protein it produces, is an early sign of fibrosis, and it's found in various tissues, including the lungs (Zhang X et al. 2013) and blood vessels (Frank M et al. 2015).<br>COL3A1 plays positive role in normal adipogenesis (Hasan M A et al. 2021) and its over-accumulation promoted adipose tissue fibrosis (Divoux A et al. 2010).
COL8A2	Type VIII collagen	FC 1.45 P=0.03	COL8A2 plays a crucial role in fibrosis, particularly in the corneal endothelium (Hwang J S et al. 2020).<br>In the vasculature, COL8A2 may play a role in cell migration and proliferation, potentially influencing vascular remodeling (Skrbic B et al. 2015; Hwang J S et al. 2020).

**ECM maintenance**			
MMP16	Matrix metallopeptidase 16	FC 1.32 P=0.006	MMP16 was considered to promote the fibrosis process (Chen Z et al. 2018).<br>High MMP-16 expression correlated significantly with capsular invasion of hepatic carcinoma (Arai I et al. 2007).
MMP19	Matrix metallopeptidase 19	FC1.3, P=0.051	MMP19 increase in hyperplastic epithelial cells surrounding fibrotic areas may have a profibrotic effect (Yu G et al. 2012).<br>MMP-19 has a potential dual role to be pro-fibrotic in the early phases post-injury, and anti-fibrotic during resolution (Jirouskova M et al. 2012).<br>MMP-19 has strong regulatory effects on the synthesis of key ECM components, on fibroblast to myofibroblast differentiation, and in migration and proliferation (Jara P et al. 2015).
MMP23B	Matrix metallopeptidase 23B	FC 1.3, P=0.02	MMP23B acts in the TGF-β1 releasing process in vivo and is likely a marker or a cause of early liver fibrosis in zebrafish (Qi F et al. 2010).
MMP27	Matrix metallopeptidase 27	FC 1.8 P=0.02	MMP-27 increased in the human endometrium at the end of the secretory phase, before menstruationmay (Talbi S et al. 2006).<br>MMP-27 overexpression in the menstrual endometrium disclosed and be involved in degrading matrix proteins and potentially promotes fibrosis (Chevronnay H P G et al. 2009).
LAMA1	Laminin	FC 6.8 P= 0.001	LAMA1 is increased in the lungs of patients with IPF and affect the production of various ECM proteins including collagen and fibronectin (Lee C-M et al. 2018).<br>LAMA1 affects how macrophages respond to TGF-β1, impacting their role in the inflammatory response and fibrotic remodeling (Lee C-M et al. 2018).<br>LAMA1 plays a role in the process of Fibroblasts transforming into myofibroblasts to produce significant amounts of ECM (Lee C-M et al. 2018).
**Regulators**			
POSTN	Periostin	FC 2.8 P= 0.0002	POSTN, linked to TGF-β signaling, promotes fibroblast proliferation, collagen deposition, and overall

			fibrosis development, contributing to the transition of fibroblasts to myofibroblasts and ECM synthesis (O'Dwyer D N and Moore B B 2017).<br>POSTN is highly expressed in the lungs of patients with idiopathic pulmonary fibrosis (IPF) and asthma, and its inhibition can suppress lung fibrosis in vivo (O'Dwyer D N and Moore B B 2017; Yamato H et al. 2021).<br>POSTN exacerbates muscle fibrosis after skeletal muscle injury, hindering muscle recovery (Hara M et al. 2018).
TGFB2	TGFB2	FC 1.4 P=0.01	TGFB2 increased in fibrotic tissues, including in the lungs and liver (Sun T et al. 2021).<br>TGFB2 activates and stimulates fibroblasts, leads to increased proliferation of fibroblasts, further contributing to the excessive ECM deposition characteristic of fibrosis (Ong C H et al. 2021).<br>TGFB2 promotes the synthesis of various ECM components, including collagen, fibronectin, and others and also inhibits the breakdown of existing ECM, further contributing to the accumulation of fibrosis tissue (Ong C H et al. 2021).<br>TGFB2, together with TGFB1 and TGFB3, can regulate the development and differentiation of white adipocytes, inhibit adipogenesis and contribute to inflammation in adipose tissue (Lee M-J 2018; Takahashi H et al. 2019).
TGFB3	TGFB3	FC 1.3 P=0.014	TGFB3 expression is increased in fibrotic tissues compared to healthy tissues, including in the liver, lungs, and skin (Sun T et al. 2021).<br>TGFB3 is involved in the initiation of liver fibrosis progression (Guo J et al. 2021).<br>TGFB3 contributes to fibrogenesis in mouse models of lung fibrosis (Sun T et al. 2021).<br>TGFB3 deficiency can lead to renal fibrosis and defective lipid metabolism (Escasany E et al. 2021).<br>TGFB3, together with TGFB1 and TGFB2, can regulate the development and differentiation of white adipocytes, inhibit adipogenesis and contribute to inflammation in adipose tissue (Lee M-J 2018; Takahashi H et al. 2019).
TGFBR1	TGFBR1	FC 1.23 P=0.049	TGF-β1 and TGFBR1 signaling are key drivers of fibroblast activation, the process where fibroblasts

			transform into myofibroblasts, which are specialized cells that produce large amounts of collagen (Ye Z and Hu Y 2021).
ADIPOR1	Adiponectin	FC -1.12 P=0.03	Adiponectin is produced in abundance in adipose tissue and serves as a critical marker of adipose tissue health and is lower in individuals with obesity (Achari A E and Jain S K 2017).<br>Adiponectin is identified as a negative regulator of tissue fibrosis (Zhang Q et al. 2024). In vitro it can inhibit fibroblast activation induced by TGF-β, LPS, and Wnt signaling pathways, as well as downregulate collagen and α-SMA gene expression (Fang F et al. 2012; Reinke L et al. 2016).
PDGFRA	PDGFRA	FC 1.25 P=0.04	PDGFRA signaling can stimulate the proliferation and migration of fibroblasts, leading to excessive amounts of extracellular matrix proteins like collagen (Paolini C et al. 2022).<br>In muscle, PDGFRA signaling can support muscle regeneration, but dysregulation can lead to fibrosis (Mueller A A et al. 2016).<br>PDGFRA signaling can interact with TGF-β to promote the fibrotic response (Branton M H and Kopp J B 1999).

References

Achari A E, Jain S K. 2017. Adiponectin, a Therapeutic Target for Obesity, Diabetes, and Endothelial Dysfunction. *International journal of molecular sciences* **18**.10.3390/ijms18061321.

Arai I, Nagano H, Kondo M, Yamamoto H, Hiraoka N, Sugita Y, Ota H, Yoshioka S, Nakamura M, Wada H et al. 2007. Overexpression of MT3-MMP in hepatocellular carcinoma correlates with capsular invasion. *Hepato-gastroenterology* **54**: 167-171.

Branton M H, Kopp J B. 1999. TGF-beta and fibrosis. *Microbes and infection* **1**: 1349-1365.10.1016/s1286-4579(99)00250-6.

Chen Z, Ding H-S, Guo X, Shen J-J, Fan D, Huang Y, Huang C-X. 2018. MiR-33 promotes myocardial fibrosis by inhibiting MMP16 and stimulating p38 MAPK signaling. *Oncotarget* **9**: 22047-22057.10.18632/oncotarget.25173.

Chevronnay H P G, Galant C, Lemoine P, Courtoy P J, Marbaix E, Henriet P. 2009. Spatiotemporal coupling of focal extracellular matrix degradation and reconstruction in the menstrual human endometrium. *Endocrinology* **150**: 5094-5105.10.1210/en.2009-0750.

Devos H, Zoidakis J, Roubelakis M G, Latosinska A, Vlahou A. 2023. Reviewing the Regulators of COL1A1. *International journal of molecular sciences* **24**.10.3390/ijms241210004.

Divoux A, Tordjman J, Lacasa D, Veyrie N, Hugol D, Aissat A, Basdevant A, Guerre-Millo M, Poitou C, Zucker J-D et al. 2010. Fibrosis in human adipose tissue: composition, distribution, and link with lipid metabolism and fat mass loss. *Diabetes* **59**: 2817-2825.10.2337/db10-0585.

Escasany E, Lanzón B, García-Carrasco A, Izquierdo-Lahuerta A, Torres L, Corrales P, Rodríguez A E R, Luis-Lima S, Álvarez C M, Ruperez F J et al. 2021. Transforming growth factor β3 deficiency promotes defective lipid metabolism and fibrosis in murine kidney. *Disease models & mechanisms* **14**.10.1242/dmm.048249.

Fang F, Liu L, Yang Y, Tamaki Z, Wei J, Marangoni R G, Bhattacharyya S, Summer R S, Ye B, Varga J. 2012. The adipokine adiponectin has potent anti-fibrotic effects mediated via adenosine monophosphate-activated protein kinase: novel target for fibrosis therapy. *Arthritis research & therapy* **14**: R229.10.1186/ar4070.

Fragiadaki M, Witherden A S, Kaneko T, Sonnylal S, Pusey C D, Bou-Gharios G, Mason R M. 2011. Interstitial fibrosis is associated with increased COL1A2 transcription in AA-injured renal tubular epithelial cells in vivo. *Matrix Biol* **30**.10.1016/j.matbio.2011.07.004.

Frank M, Albuisson J, Ranque B, Golmard L, Mazzella J-M, Bal-Theoleyre L, Fauret A-L, Mirault T, Denarié N, Mousseaux E et al. 2015. The type of variants at the COL3A1 gene associates with the phenotype and severity of vascular Ehlers-Danlos syndrome. *Eur J Hum Genet* **23**.10.1038/ejhg.2015.32.

Guo J, Liu W, Zeng Z, Lin J, Zhang X, Chen L. 2021. Tgfb3 and Mmp13 regulated the initiation of liver fibrosis progression as dynamic network biomarkers. *Journal of cellular and molecular medicine* **25**: 867-879.10.1111/jcmm.16140.

Hara M, Yokota K, Saito T, Kobayakawa K, Kijima K, Yoshizaki S, Okazaki K, Yoshida S, Matsumoto Y, Harimaya K et al. 2018. Periostin Promotes Fibroblast Migration and Inhibits Muscle Repair After Skeletal Muscle Injury. *J Bone Joint Surg Am* **100**.10.2106/jbjs.17.01230.

Hasan M A, Martin P E, Shu X, Patterson S, Bartholomew C. 2021. Type III Collagen is Required for Adipogenesis and Actin Stress Fibre Formation in 3T3-L1 Preadipocytes. *Biomolecules* **11**.10.3390/biom11020156.

Hua X, Wang Y-Y, Jia P, Xiong Q, Hu Y, Chang Y, Lai S, Xu Y, Zhao Z, Song J. 2020. Multi-level transcriptome sequencing identifies COL1A1 as a candidate marker in human heart failure progression. *BMC medicine* **18**: 2.10.1186/s12916-019-1469-4.

Hwang J S, Ma D J, Choi J, Shin Y J. 2020. COL8A2 Regulates the Fate of Corneal Endothelial Cells. *Invest Ophthalmol Vis Sci* **61**.10.1167/iovs.61.11.26.

Jara P, Calyeca J, Romero Y, Plácido L, Yu G, Kaminski N, Maldonado V, Cisneros J, Selman M, Pardo A. 2015. Matrix metalloproteinase (MMP)-19-deficient fibroblasts display a profibrotic phenotype. *American journal of physiology Lung cellular and molecular physiology* **308**: L511-522.10.1152/ajplung.00043.2014.

Jirouskova M, Zbodakova O, Gregor M, Chalupsky K, Sarnova L, Hajduch M, Ehrmann J, Jirkovska M, Sedlacek R. 2012. Hepatoprotective effect of MMP-19 deficiency in a mouse model of chronic liver fibrosis. *PloS one* **7**: e46271.10.1371/journal.pone.0046271.

Lee C-M, Cho S J, Cho W-K, Park J W, Lee J-H, Choi A M, Rosas I O, Zheng M, Peltz G, Lee C G et al. 2018. Laminin α1 is a genetic modifier of TGF-β1-stimulated pulmonary fibrosis. *JCI insight* **3**.10.1172/jci.insight.99574.

Lee M-J. 2018. Transforming growth factor beta superfamily regulation of adipose tissue biology in obesity. *Biochimica et biophysica acta Molecular basis of disease* **1864**: 1160-1171.10.1016/j.bbadis.2018.01.025.

Mueller A A, Velthoven C T v, Fukumoto K D, Cheung T H, Rando T A. 2016. Intronic polyadenylation of PDGFRα in resident stem cells attenuates muscle fibrosis. *Nature* **540**: 276-279.10.1038/nature20160.

Nassabeh N. 2009. Elucidating the origins of lung fibrosis. *Nature Reviews Rheumatology* **5**: 467-467.10.1038/nrrheum.2009.153.

O'Dwyer D N, Moore B B. 2017. The role of periostin in lung fibrosis and airway remodeling. *Cell Mol Life Sci* **74**.10.1007/s00018-017-2649-z.

Ong C H, Tham C L, Harith H H, Firdaus N, Israf D A. 2021. TGF-β-induced fibrosis: A review on the underlying mechanism and potential therapeutic strategies. *European journal of pharmacology* **911**: 174510.10.1016/j.ejphar.2021.174510.

Pan X, Chen X, Ren Q, Yue L, Niu S, Li Z, Zhu R, Chen X, Jia Z, Zhen R et al. 2022. Single-cell transcriptomics identifies Col1a1 and Col1a2 as hub genes in obesity-induced cardiac fibrosis. *Biochemical and biophysical research communications* **618**: 30-37.10.1016/j.bbrc.2022.06.018.

Paolini C, Agarbati S, Benfaremo D, Mozzicafreddo M, Svegliati S, Moroncini G. 2022. PDGF/PDGFR: A Possible Molecular Target in Scleroderma Fibrosis. *International journal of molecular sciences* **23**.10.3390/ijms23073904.

Qi F, Song J, Yang H, Gao W, Liu N-a, Zhang B, Lin S. 2010. Mmp23b promotes liver development and hepatocyte proliferation through the tumor necrosis factor pathway in zebrafish. *Hepatology (Baltimore, Md)* **52**: 2158-2166.10.1002/hep.23945.

Reinke L, Lam A P, Flozak A S, Varga J, Gottardi C J. 2016. Adiponectin inhibits Wnt co-receptor, Lrp6, phosphorylation and β-catenin signaling. *Biochemical and biophysical research communications* **470**: 606-612.10.1016/j.bbrc.2016.01.097.

Skrbic B, Engebretsen K V T, Strand M E, Lunde I G, Herum K M, Marstein H S, Sjaastad I, Lunde P K, Carlson C R, Christensen G et al. 2015. Lack of collagen VIII reduces fibrosis and promotes early mortality and cardiac dilatation in pressure overload in mice. *Cardiovasc Res* **106**.10.1093/cvr/cvv041.

Sun K, Li X, Scherer P E. 2023. Extracellular Matrix (ECM) and Fibrosis in Adipose Tissue: Overview and Perspectives. *Comprehensive Physiology* **13**: 4387-4407.10.1002/cphy.c220020.

Sun T, Huang Z, Liang W-C, Yin J, Lin W Y, Wu J, Vernes J-M, Lutman J, Caplazi P, Jeet S et al. 2021. TGFβ2 and TGFβ3 isoforms drive fibrotic disease pathogenesis. *Science translational medicine* **13**.10.1126/scitranslmed.abe0407.

Takahashi H, Alves C R R, Stanford K I, Middelbeek R J W, Nigro P, Ryan R E, Xue R, Sakaguchi M, Lynes M D, So K et al. 2019. TGF-β2 is an exercise-induced adipokine that regulates glucose and fatty acid metabolism. *Nature metabolism* **1**: 291-303.10.1038/s42255-018-0030-7.

Talbi S, Hamilton A E, Vo K C, Tulac S, Overgaard M T, Dosiou C, Shay N L, Nezhat C N, Kempson R, Lessey B A et al. 2006. Molecular phenotyping of human endometrium distinguishes menstrual cycle phases and underlying biological processes in normo-ovulatory women. *Endocrinology* **147**: 1097-1121.10.1210/en.2005-1076.

Tsitoura E, Trachalaki A, Vasarmidi E, Mastrodemou S, Margaritopoulos G A, Kokosi M, Fanidis D, Galaris A, Aidinis V, Renzoni E et al. 2021. Collagen 1a1 Expression by Airway Macrophages Increases In Fibrotic ILDs and Is Associated With FVC Decline and Increased Mortality. *Frontiers in immunology* **12**: 645548.10.3389/fimmu.2021.645548.

Yamato H, Kimura K, Fukui E, Kanou T, Ose N, Funaki S, Minami M, Shintani Y. 2021. Periostin secreted by activated fibroblasts in idiopathic pulmonary fibrosis promotes tumorigenesis of non-small cell lung cancer. *Sci Rep* **11**.10.1038/s41598-021-00717-5.

Ye Z, Hu Y. 2021. TGF- β1: Gentlemanly orchestrator in idiopathic pulmonary fibrosis (Review). *International journal of molecular medicine* **48**.10.3892/ijmm.2021.4965.

Yu G, Kovkarova-Naumovski E, Jara P, Parwani A, Kass D, Ruiz V, Lopez-Otín C, Rosas I O, Gibson K F, Cabrera S et al. 2012. Matrix metalloproteinase-19 is a key regulator of lung fibrosis in mice and humans. *American journal of respiratory and critical care medicine* **186**: 752-762.10.1164/rccm.201202-0302OC.

Zhang Q, Lu C, Lu F, Liao Y, Cai J, Gao J. 2024. Challenges and opportunities in obesity: the role of adipocytes during tissue fibrosis. *Frontiers in endocrinology* **15**: 1365156.10.3389/fendo.2024.1365156.

Zhang X, Liu H, Hock T, Thannickal V J, Sanders Y Y. 2013. Histone deacetylase inhibition downregulates collagen 3A1 in fibrotic lung fibroblasts. *Int J Mol Sci* **14**.10.3390/ijms141019605.
